# Delivery type not associated with global methylation at birth

**DOI:** 10.1186/1868-7083-4-8

**Published:** 2012-06-09

**Authors:** Shama Virani, Dana C Dolinoy, Sindhu Halubai, Tamara R Jones, Steve E Domino, Laura S Rozek, Muna S Nahar, Vasantha Padmanabhan

**Affiliations:** 1 Department of Environmental Health Sciences, The University of Michigan, Room 1138, 300 N. Ingalls Building, Ann Arbor, MI, 48109-0404, USA; 2 Department of Pediatrics, The University of Michigan, Ann Arbor, MI, USA; 3 Department of Obstetrics and Gynecology, The University of Michigan, Ann Arbor, MI, USA; 4 Department of Otolaryngology, The University of Michigan, Ann Arbor, MI, USA

**Keywords:** DNA methylation, Delivery type, Epigenetics, Infant

## Abstract

****Background**:**

Birth by cesarean delivery (CD) as opposed to vaginal delivery (VD) is associated with altered health outcomes later in life, including respiratory disorders, allergies and risk of developing type I diabetes. Epigenetic gene regulation is a proposed mechanism by which early life exposures affect later health outcomes. Previously, type of delivery has been found to be associated with differences in global methylation levels, but the sample sizes have been small. We measured global methylation in a large birth cohort to identify whether type of delivery is associated with epigenetic changes.

****Methods**:**

DNA was isolated from cord blood collected from the University of Michigan Women’s & Children Hospital and bisulfite-converted. The Luminometric Methylation Assay (LUMA) and LINE-1 methylation assay were run on all samples in duplicate.

****Results**:**

Global methylation data at CCGG sites throughout the genome, as measured by LUMA, were available from 392 births (52% male; 65% CD), and quantitative methylation levels at LINE-1 repetitive elements were available for 407 births (52% male; 64% CD). LUMA and LINE-1 methylation measurements were negatively correlated in this population (Spearman’s r = −0.13, *p* =0.01). LUMA measurements were significantly lower for total CD and planned CD, but not emergency CD when compared to VD (median VD = 74.8, median total CD = 74.4, *p* = 0.03; median planned CD = 74.2, *p* = 0.02; median emergency CD = 75.3, *p* = 0.39). However, this association did not persist when adjusting for maternal age, maternal smoking and infant gender. Furthermore, total CD deliveries, planned CD and emergency CD deliveries were not associated with LINE-1 measurements as compared to VD (median VD = 82.2, median total CD = 81.9, *p* = 0.19; median planned CD = 81.9, *p* = 0.19; median emergency CD = 82.1, *p* = 0.52). This lack of association held when adjusting for maternal age, maternal smoking and infant gender in a multivariable model.

****Conclusions**:**

Type of delivery was not associated with global methylation in our population, even after adjustment for maternal age, maternal smoking, and infant gender. While type of birth may be associated with later health outcomes, our data suggest that it does not do so through changes in global genomic methylation.

## **Background**

Infant stress during labor is advantageous in preparation for extrauterine life; however, the stress encountered by infants varies vastly between delivery types. The progression of labor involves a surge in stress hormones, including catecholamines and cortisol, in the infant to promote lung maturity for gas exchange, increase blood flow, activate the central nervous system and mobilize fuel [1,2]. This high release of stress hormones also triggers a cascade of hormones and cytokines involved in inflammatory defense pathways [1,3]. Uncomplicated vaginal deliveries (VDs) undergo a normal progression of labor in which the infant experiences these critical changes; however, elective cesarean deliveries (CDs) do not, and infants born via this CD typically lack the necessary surges of hormone release. In emergency CDs, labor may be incomplete and the full release of hormones may not occur. Comparison of these different delivery types shows that infants born via VD have higher levels of hormone release [1,2,4-6] as well as a greater number of intestinal flora [7] and higher immune response [8] than those born through elective CD. Emergency CD infants have higher hormone levels than those born via elective CD, but lower than those born via VD [9].

Surges of stress hormones during labor progress development of essential pathways that will enable the infant to survive outside the womb, thus implicating changes in the genome in response to stress. However, these changes may not occur if the infant does not undergo such stress, such as during a CD. The increasing number of CDs has led to scrutiny of the impact that type of delivery has on infant health and predisposition to disease outcomes. Birth via CD is associated with a higher risk of developing diseases such as allergic rhinoconjunctivitis and asthma [10,11], type 1 diabetes [12], respiratory disorders [13] and pediatric Crohn’s disease [14].

The molecular mechanisms associated with these phenomena remain unknown; however, emerging evidence suggests that when an infant does not develop essential defense mechanisms, he or she may be predisposed to developing disease later on in life. The developmental origins of health and disease (DOHaD) hypothesis posits that increased susceptibility to disease following early life experiences is shaped by epigenetic modifications such as DNA methylation and chromatin modifications [15]. Epigenetics allows for adaptive responses of the genome to an ever-changing environment by modifying transcriptional regulation of gene expression. These adaptations are achieved in response to developmental stage, environmental exposures and cell differentiation. Due to the pliability of the epigenome in response to surroundings, such as delivery environment, altered fetal or neonatal epigenetic modifications may play a role in later-life disease susceptibility [16-18]. While many types of epigenetic modifications occur, DNA methylation is the most extensively studied. Because DNA methylation is inherited in somatic cells, early epigenetic events can have lasting effects on gene expression, which may in turn provide the molecular basis for susceptibility to disease later in life [17].

A disease outcome likely results from aberrant activity of many different molecular pathways, and thus it is likely that epigenetic modifications occur at multiple genes or genic regions. Measurement of global DNA methylation patterns is a practical means of identifying differential epigenetic effects in neonates. Furthermore, recent research has shown differential global methylation between CD and VD infants, although the sample size was small [19].

The objectives of this study are to (1) confirm the apparent difference in global DNA methylation between infants born via CD as compared to VD in white blood cells from cord blood in a large population, using two separate global DNA methylation measurements and (2) determine if methylation differences are associated with type of CD (for example, scheduled versus emergency) as compared to VD.

## **Methods**

### **Study population**

IRB approval was obtained for the purpose of collecting excess cord blood samples from our study population, which consisted of 408 infants (> 37 weeks) delivered at the University of Michigan Women’s & Children Hospital. Only pregnancies occurring between 8 a.m. and 2 p.m. were included to provide more time for sample processing, and included scheduled and emergency CDs as well as VDs. Because this study collected excess clinical samples, samples were obtained from all deliveries during this time period. Samples were collected with few exclusion criteria, only requiring singleton pregnancy and clinically excess tissue available immediately after delivery in order to maximize sample quality. After delivery of the placenta, the umbilical cord was clamped, washed thoroughly to prevent maternal blood contamination, and whole venous blood was drawn via venipuncture into PAX gene Blood DNA tubes (Qiagen Inc., Valencia, CA), kept on ice and transported to the laboratory.

### **DNA isolation and bisulfite conversion**

Genomic DNA was extracted using the PAX gene Blood DNA kit (PreAnalytiX/Qiagen, Hombrechtikon, Switzerland). Leukocyte DNA was isolated following the manufacturer’s protocol with some minor modifications for dense blood. For complete digestion of dense samples, the initial 65°C incubation period was increased from 10 min to 1 h. The final incubation of the solution was repeated a second time in order to completely dissolve the DNA containing pellet in elution buffer. Both the quality and quantity of the extracted DNA were assessed using a ND1000 spectrophotometer (NanoDrop Technology, Wilmington, DL). For bisulfite conversion, the addition of sodium bisulfite selectively deaminates unmethylated cytosine nucleotides into uracil whereby a subsequent polymerase chain reaction (PCR) incorporates thymine, leaving methylated cytosines unchanged. Complete bisulfite modification of genomic DNA results in methylation-dependent genome-wide alterations in DNA sequences. Approximately 1 μg of genomic cord blood DNA was bisulfite converted using the EpiTect Bisulfite Kit (Qiagen Inc., Valencia, CA) and QIAcube® purification system.

### **LUMA and LINE-1 assays**

The Luminometric Methylation Assay (LUMA) is a restriction enzyme-based procedure that detects methylation at 5’-CCGG-3’ sequences throughout the genome, irrespective of methylation at repetitive elements. The procedure for LUMA analysis via pyrosequencing has been previously described [20,21]. Briefly, approximately 5 units of methylation-insensitive *MspI* (Biolabs) and of methylation-sensitive *HpaII* (Biolabs) enzymes were separately added to 300 ng of genomic DNA along with the internal standard *EcoRI* (Biolabs) enzyme and 10× buffer.

Tango™with BSA (Fermentes) was used for differential digestion. Samples were incubated at 37°C for 4 h. Twenty μl of annealing buffer was added prior to pyrosequencing in AQ mode using the SNP PyroMarkMD software with the following dispensation order: GTGTCACATGTGTG. The ratio of normalized product signal between methylation-sensitive and -insensitive digestions from an individual sample was measured to calculate global DNA methylation using the following equation: 1 − [(HpaII/EcoRI)/(MspI/EcoRI)] × 100. Individual samples were run in duplicate along with lowly and highly methylated human genomic standards (EpigenDx).

The LINE-1 assay interrogates promoter DNA methylation of repetitive elements, specifically long interspersed (LI) retrotransposons, throughout the genome [22]. Fifty ng/μl of bisulfite converted DNA was used in a PCR mix containing water, HotStarTaq master mix (Qiagen), (1 pmol) LINE-1 forward and (0.5 pmol) LINE-1 biotin-labeled reverse primers, followed by amplification via specific PCR cycling parameters: 95°C for 14.5 min (Hot Start), 95°C for 30 s, 58°C for 30 s, 72°C for 30 s at 45 cycles. We also included bisulfite-converted DNA from lowly and highly methylated controls. Amplified products were verified using gel electrophoresis. LINE-1 amplified products were analyzed in duplicate using the PyroMark™Q96 MD Pyrosequencing System (Pyrosequencing, Inc., Westborough, MA) and a predetermined sequence-to-analyze run. Approximately 15 μl of LINE-1 PCR product was added with LINE-1 sequencing primers. Methylation analysis by pyrosequencing was conducted as previously described [23]. The following modified primers (Invitrogen) [22] were used for the complete assessment: LINE-1 forward primer: 5’-TTG AGT TAG GTG TGG GAT ATA GTT-3’; reverse primer: 5’-CAA AAA ATC AAA AAA TTC CCT TTC C-3’; sequencing primer: 5’-AGG TGT GGA TAT AGT-3’; sequence to analyze: TT[T/C]GTGGTG[T/C]GT[T/C]GTTTTTTAAGT[T/C]GGTTTGAAAAG. The PyroMark Q-CpG software computes the ratio of cytosine and thymine signal intensity at each of the four CpG sites in this assay, referenced as site 1, 2, 3 and 4, and outputs a % methylation value for DNA methylation analysis of LINE-1 global and gene-specific assays.

### **Statistical analysis**

Duplicate LUMA and LINE-1 global methylation measurements were averaged for all analyses, and duplicates resulting in a greater than 15% difference were deleted before statistical analysis, leading to 16 subjects deleted for LUMA and 1 deleted for LINE-1. Due to departures from normality, data are expressed as median values and ranges. Bivariate nonparametric tests (Wilcoxon rank sum test and Kruskal-Wallis test) were used to test the association between global methylation (LUMA and LINE-1) and covariates (type of delivery, infant gender, maternal age, maternal smoking). Significance of correlations between covariates was tested using Spearman’s correlation. Generalized linear models were used to test the association between type of deliveries with each methylation measurement, adjusting for maternal age, maternal smoking status and infant gender. Mixed effects models were used to test the association between each specific LINE-1 site (site 1, site 2, site 3 and site 4) to account for covariance between measurements. Statistical significance was set at *p* < 0.05. Generalized linear models were run in R version 2.12.0 (R Development Core Team, 2010), and mixed effects models were run in SAS 9.2 (Cary, NC).

## **Results**

### **Descriptive statistics**

Data were available for 408 subjects. Thirty-six percent were VDs, while 64% delivered via CD. Of the CD deliveries, 80% were planned CD deliveries, and 20% were emergency CD deliveries. Maternal age ranged from 18–48 years, with approximately equal male and female infants (52%, 48%, respectively). The majority of mothers did not have a history of smoking (60%), while 11% were past smokers and 6% were current smokers; for 93 of the 408 subjects, smoking status was unknown (Table 1). There were significant correlations between age and type of delivery as well as smoking and type of delivery; however, the magnitude of the correlations was low (*rho* = 0.2, 0.1, *p* < 0.001, *p* = 0.03, respectively).

**Table 1:** Global methylation by delivery characteristics

	**LUMA (%)**			**LINE1-1 (%)**			**LINE1-2 (%)**		**LINE1-3 (%)**		**LINE1-4 (%)**		**Mean LINE1**	
*Features*	*No.*	*Median (range)*	*Mean*	*No.*	*Median (range)*	*Mean*	*Median (range)*	*Mean*	*Median (range)*	*Mean*	*Median (range)*	*Mean*	*Median (range)*	*Mean*
**Overall**	392	74.5 (46.3-83.6)	73.8	407	85.1 (72.7-98.7)	84.5	82.7 (74.1 95.0)	82.2	81.6 (71.3-98.7)	81.4	78.6 (71.8-97.0)	78.4	82.0 (73.6-97.4)	81.6
Vaginal	254	74.4 (46.3-81.6)	73.5	262	85.1 (72.7-90.6)	84.3	82.6 (74.1-87.2)	82.2	81.4 (71.3-90.6)	81.2	78.4 (71.9-90.5)	78.3	81.9 (74.9-85.7)	81.5
Planned CD	201	74.2 (46.3-81.6)	73.4	207	85.1 (72.7-90.4)	84.4	82.6 (75.5-87.2)	82.2	81.3 (71.3-90.6)	81.2	78.4 (71.9-89.2)	78.3	81.9 (74.9-85.6)	81.5
Planned CD	53	75.3 (56.6-79.2)	73.9	55	85.0 (76.2-90.6)	84.3	82.8 (74.1-85.6)	82.1	81.7 (74.3-85.9)	81.4	78.4 (72.0-90.5)	78.5	82.1 (76.1-85.7)	81.6
Emergency CD	138	74.8 (56.4-83.6)	74.5	145	85.3 (73.8-98.7)	84.7	82.7 (74.3-95.0)	82.3	82.1 (71.8-98.7)	81.7	78.9 (71.8-97.0)	78.6	82.2 (73.6-97.4)	81.8
**Infant gender**														
Male	203	74.6 (46.3-83.6)	73.5	213	85.0 (73.8-98.7)	84.3	82.6 (75.5-90.0)	82.2	81.5 (71.3-98.7)	81.5	78.7 (71.9-97.0)	78.7	82 (76.3-97.4)	81.7
Female	188	74.4 (55.5-83.6)	74.2	193	85.3 (72.7-90.7)	84.7	82.8 (74.1-87.2)	82.3	81.7 (71.8-85.5)	81.3	78.5 (71.8-83.9)	78.2	82.1 (73.6-85.6)	81.62
**Smoking**														
Never	234	74.6 (46.3-83.6)	73.8	243	85.1 (72.7-90.4)	84.3	82.6 (75.5-87.6)	82.1	81.3 (71.3-86.7)	81.2	78.4 (71.8-90.5)	78.2	81.9 (75.5-85.6)	81.4
Past	44	75.1 (59.3-81.0)	74.1	45	85.0 (73.8-91.8)	84.8	82.7 (74.1-85.6)	82.1	81.5 (74.5-89.3)	81.1	78.4 (74.6-83.9)	78.2	82.2 (76.1-87.3)	81.6
Current	24	73.9 (64.7-81.6)	73.9	26	85.3 (78.3-98.7)	85.6	82.5 (75.9-95.0)	82.5	81.8 (75.0-98.7)	82.4	79.7 (75.8-97.0)	80.3	82.5 (77.4-97.4)	82.7
**Age**														
18-27	111	75.0 (59.3-83.6)	74.5	117	84.8 (72.8-98.7)	83.9	82.6 (74.1-95.0)	82.1	82.0 (71.8-98.7)	81.5	78.7 (71.8-97.0)	78.3	82.0 (73.6-97.4)	81.5
28-37	232	74.3 (46.3-81.6)	73.6	241	85.4 (72.7-91.8)	84.8	82.8 (75.7-87.6)	82.3	81.5 (71.3-90.6)	81.4	78.7 (72.4-90.5)	78.6	82.1 (75.5-87.3)	81.8
38-48	49	74.5 (56.2-79.8)	73.7	49	84.7 (77.9-89.8)	84.3	82.4 (76.0-85.3)	82	81.4 (76.2-85.9)	81.2	77.3 (71.9-89.2)	77.8	81.5 (76.3-85.0)	81.3

### **Global methylation at CCGG sites (LUMA)**

Among the sample population, the median LUMA measurement was 74.5% (46.3-83.6), and mean LUMA measurement was 73.8% (Table 1). In comparing VD with total CD (planned and emergency combined), CD deliveries had significantly lower LUMA methylation than VD (median CD = 74.4, median VD = 74.8, *p* = 0.03); however, this association did not persist after adjustment for maternal age, maternal smoking and infant gender (Table 2).

**Table 2:** Association between all types of deliveries and global DNA methylation measurements Association between delivery types and global DNA methylation

	**VD**^**a**^	**Total CD**^**b**^		**Planned CD**		**Emergency CD**		
	**β (95% CI)**	**β (95% CI)**	***p-*value**	**β (95% CI)**	***p-*value**	**β (95% CI)**	***p*-value**	***p*-value for trend**^**d**^
*MeanLUMA*	*n=138*	*n=254*		*n=201*		*n=53*		
Crude	1.00	−1.01 (−1.90, -0.12)	0.03	−1.12 (−2.05, -0.19)	0.02	−0.60 (−1.96, 0.77)	0.39	0.12
Model 1^c^	1.00	−0.91 (−1.99, 0.17)	0.1	−1.00 (−2.11, 0.12)	0.08	−0.53 (−2.16, 1.10)	0.54	0.28
*MeanLINE-1*	*n=145*	*n=262*		*n=207*		*n=55*		
Crude	1.00	−0.31 (−0.78, 0.16)	0.19	−0.33 (−0.82, 0.16)	0.19	−0.24 (−0.95, 0.48)	0.52	0.32
Model 1^ce^	1.00	−0.31 (−0.78, 0.15)	0.27	−0.32 (−0.89, 0.25)	0.28	−0.30 (−1.14, 0.54)	0.48	0.30

^a^Reference category.

^b^Including both planned and emergency CD.

^c^Adjusted for maternal smoking, maternal age and infant gender.

^d^Trend among VD, planned CD and emergency CD.

^e^Current smokers *p*-value < 0.05.

When comparing types of CDs with VD, LUMA measurements in infants born via planned CS were significantly lower than in infants born through VD [median VD = 74.8 (56.4-83.6), median planned CD = 74.2 (46.3-81.6), *p* = 0.02]. There was no difference in methylation between VD and emergency CDs [median emergency CD = 75.3 (56.6-79.2), *p* = 0.39]. When adjusting for maternal age, maternal smoking and infant gender, LUMA methylation measurements in infants born via planned CD were still lower than those found in infants born via VD; however, this result was not significant (*p* = 0.08) in the multivariable model. There was no difference in methylation between emergency CD and total CD with VD infants after adjusting for covariates (*p* = 0.28) (Table 2). Point estimates for type of deliveries were almost as large as before adjustment, but were no longer significant after adjustment for maternal age, maternal smoking and infant gender. The magnitude of effect for the covariates may explain this observation (Table 3).

**Table 3:** Magnitude of effects for covariates

	**Mean LUMA**		**Mean LINE-1**	
	**β (95% CI)**	***p*-value**	**β (95% CI)**	***p*-value**
***Maternal age***	−0.04 (−0.13, 0.05)	0.42	−0.04 (−0.08, 0.01)	0.13
***Infant gender***^***a***^	0.68 (−0.30, 1.66)	0.17	0.06 (−0.44, 0.57)	0.80
***Former smoker***^***b***^	0.24 (−1.20,1.68)	0.74	0.17 (−0.58, 0.92)	0.66
_***Current smoker***_^***b***^	0.15 (−1.71, 2.01)	0.87	1.12 (0.18, 2.07)	0.02*

^a^Reference category = male.

^b^Reference category = never smokers.

*p-value < 0.05.

### **Methylation at LINE-1 repetitive elements**

The median LINE-1 methylation for the sample population was 82.0% (range: 73.6-97.4), and the mean was 81.6% (Table 1). In comparing total CDs with VD, there was no difference found in LINE-1 methylation (*p* = 0.19). This result did not change after adjustment for maternal age, maternal smoking and infant gender (*p* = 0.27) (Table 2). There was also no difference in mean LINE-1 methylation in infants born via planned CD [median = 81.9 (74.9-85.6), *p* = 0.19] and via emergency CD [median = 82.1 (76.1-85.7), *p* = 0.52] compared to those born through VD [median = 82.2 (73.6-97.4)] (Table 2). These results did not change after adjustment for maternal age, maternal smoking and infant gender. Point estimates for adjusted and unadjusted models were similar, implicating that these covariates may influence LINE-1 methylation (Table 3).

Comparing individual sites within LINE-1, sites 2, 3 and 4 all had significantly lower methylation than site 1 (*p =* < 0.001 for all). Type of delivery was not associated with methylation of any individual LINE-1 site (Table 4). These results did not change after adjustment for maternal age, maternal smoking and infant gender. For mean LINE-1 and all individual LINE-1 sites, infants born to mothers who reported current smoking had significantly higher methylation compared to infants born to mothers without a history of smoking (*p* = 0.02). This was seen in both crude and adjusted models of smoking.

**Table 4:** Association between type of delivery and Line-1 sites

	VD^a^	Total CD^b^		Planned CD		Emergency CD		
	**β (95% CI)**	**β (95% CI)**	***p-value***	**β (95% CI)**	***p-value***	**β (95% CI)**	***p-value***	***p-value for trend***^d^
*Crude*	*n=145*	*n=262*		*n=207*		*n=55*		
Site 1	1.00	1.00		1.00		1.00		
Site 2	1.00	0.26 (−0.31, 0.83)	0.37	0.27 (−0.33, 0.86)	0.38	0.22 (−0.64, 1.09)	0.62	0.47
Site 3	1.00	−0.06 (−0.51, 0.62)	0.84	−0.11 (−0.71, 0.48)	0.71	0.16 (−0.71, 1.02)	0.72	0.88
Site 4	1.00	0.05 (−0.52, 0.61)	0.87	−0.02 (−0.57, 0.62)	0.94	0.31 (−0.56, 1.18)	0.48	0.60
*Adjusted*^*c*^								
Site 1	1.00	1.00		1.00		1.00		
Site 2	1.00	0.20 (−0.46, 0.86)	0.55	0.22 (−0.47, 0.90)	0.54	0.14 (−0.87, 1.15)	0.79	0.66
Site 3	1.00	−0.37 (−0.29, 1.03)	0.27	−0.43 (−1.11, 0.26)	0.23	−0.16 (−1.17, 0.85)	0.75	0.49
Site 4	1.00	−0.11 (−0.77, 0.54)	0.73	−0.19 (−0.88, 0.50)	0.59	0.17 (−0.84, 1.18)	0.74	0.95

Mixed effects model.

^a^Reference category.

^b^Including both planned and emergency *CD.*

^c^Adjusted for maternal smoking, maternal age and infant gender.

^d^Trend among *VD*, Planned *CD* and emergency *CD.*

LUMA and LINE-1 measurements were negatively correlated (Spearman’s r = −0.13, *p* = 0.01). The coefficient of variation for LUMA was 5.86%, and the coefficient of variation for LINE-1 was 2.82%. When statistical analyses were restricted to individuals with both measures (*n* = 392) similar results were observed (data not shown).

## **Discussion**

It is crucial to study epigenetic modifications in an early life context to better understand their influence on an individual’s risk of developing disease later in life. Infant delivery is a prominent early life event involving major hormonal changes. In VDs, infants undergo high stress levels, as indicated by the elevation of stress hormones [24] and increase in immune function [8] as compared to those delivered via CD. This sudden increase in stress and immune activation may correspond to a change in environment, potentially inducing adaptive modifications of the epigenome. This study looked at methylation differences resulting from type of delivery to determine if global DNA and repetitive element methylation changes could be the molecular mechanism for disease susceptibility later in life. Global methylation levels can be measured in multiple ways, and in our study, global methylation at CCGG was negatively correlated with global repetitive element methylation at LINE-1 sites. LINE-1 measurements of global repetitive element methylation provide an index of genomic instability. The LINE-1 assay measures methylation of the LINE-1 retrotransposons that make up approximately 17% of the human genome; however, only about 3,000-5,000 of these elements are actively transposing within the genome [25]. Methylation of LINE-1 elements is crucial to suppress their retrotransposition, minimizing insertion into essential genes or promoters that would cause genomic instability. LINE-1 methylation measurements are a general indication of the amount of genomic instability that may occur when global methylation levels are low. Our results show no significant difference in LINE-1 mean methylation or LINE-1 site-specific methylation between CD and VD births. When CDs were further categorized into planned and emergency CD, no significant differences in LINE-1 methylation levels were observed, even when adjusting for infant gender, maternal smoking and maternal age.

A second option for global methylation measurement is the LUMA method [20,21]. LUMA measures methylation at CCGG sites throughout the entire genome regardless of location, illustrating global methylation at these sites. The amount of LUMA methylation may have implications for levels of gene expression across various cellular pathways and is often indicative of pathological processes [20,21]. Our delivery type analysis indicates that LUMA measurements are significantly lower for CDs than VDs, although this association does not persist after adjusting for maternal age, maternal smoking and infant gender. In further categorizing CDs, there are no differences between emergency CD and VD, but planned CDs had significantly lower methylation compared to VDs. This association, however, does not persist when adjusting for maternal age, maternal smoking and infant gender. Although the association was attenuated, the pattern of lower global methylation in planned CD infants is seen in both the crude and adjusted models, indicating that a significant association may be achieved with a larger sample size for subgroup analyses. However, the crude analysis for the LUMA assay may be the most appropriate for understanding global methylation as a result of delivery type. If maternal age or smoking is the driving force behind this association, then global methylation might be an intermediate variable between these predictors and type of delivery, which may attenuate some of the effect for LUMA. Even if smoking or maternal age is upstream from LUMA with respect to the causal pathway, both are likely to have multiple mechanisms of action, including methylation and other non-epigenetic mechanisms*.* Additionally, birth weight could be added into the model. A recently published study reported that birth weight and prematurity are associated with lower LINE-1 methylation [26].

A previous study conducted by Schlinzig et al. [19] analyzing the relationship between type of delivery and LUMA measurements reported significantly higher methylation in infants delivered by elective CD than those by VD, although this association was lost 3–5 days after birth. Our results of lower methylation in planned CDs conflict with the previous study’s findings of higher methylation in elective CDs. However, specific aspects of the individual study designs that differ may explain the discrepancy. Schlinzig et al. used a population of 37 infants from Stockholm, Sweden, whereas we assessed a population of 408 infants from Ann Arbor, Michigan. Population differences, sampling variance or both may play a role in the distinctive findings. With our large sample size we provide adequate power to our results. 

The LINE-1 mean and site-specific models adjusted for maternal age, maternal smoking and infant gender consistently indicate that current maternal smokers deliver infants with significantly higher methylation than those born from mothers who never smoked, although the effect is modest. This association between smoking and LINE-1 methylation is significant in both crude and adjusted models. These results indicate that smoking has an effect on LINE-1 methylation as established by previous literature, although the direction of effect has remained inconclusive [27,28]. In our study, LINE-1 methylation was higher for infants born from current smokers as compared to infants born from non-smokers, although smoking did not differ by delivery type. A limitation in our study is the low prevalence of current smoking in this sample of women (6%). The prevalence of current smoking in the US population is around 20% [29]. It is possible that our subgroup of smokers was too small to detect an association between smoking and methylation due to type of delivery.

## **Conclusions**

In summary, no differences in global DNA or repetitive element methylation of cord-blood derived leukocytes were observed in infants delivered via planned CD or emergency CD as compared to those delivered vaginally. A strength of this study is a strong foundation for future investigations, namely the large sample size, categorization of CDs into planned and emergency types, and the use of two separate global methylation measurement techniques. This approach not only provides power to the study, but also takes into account the different types of stress from delivery on the infant. A limitation of this study is the lack of longitudinal post-delivery epigenetic measurements. Methylation patterns may change over time, and such data would be useful for future epigenetic epidemiology study considerations. The evaluation of methylation profiles should be investigated across multiple time points, as it is possible the type-of-delivery effects on the epigenome manifest later in life. Although this study provides negative findings when assessing *global* genomic methylation patterns, it does not discount the potential for delivery type to affect the infant epigenome in a gene-specific manner. Future studies should focus on candidate gene methylation changes in pathways involved in diseases associated with CDs.

## Abbreviations

(LUMA): luminometric methylation assay; (CD): cesarean delivery; (VD): vaginal delivery; (DOHaD): developmental origins of health and disease.

## **Competing interests**

The authors declare that they have no competing interests.

## **Authors’ contributions**

VP serves as the principal investigator of this birth cohort and the IRB protocol. VP, SED and SH identified subjects and collected cord blood immediately after birth. VP, DCD and LSR conceived of the study question and assisted in data analysis, interpretation and conclusions. SV analyzed data and drafted the manuscript. DCD and LSR supervised laboratory and statistical analyses. TRJ isolated DNA and conducted LINE-1 methylation assays. MSN conducted LINE-1 and LUMA assays. All co-authors assisted in data interpretation and manuscript revisions. All authors read and approved the final manuscript.
